# The Prognostic Significance of Resting Regional Left Ventricular Function in Patients With Varying Degrees of Myocardial Ischemia

**DOI:** 10.4021/cr240w

**Published:** 2014-01-02

**Authors:** Niamh M. Kilcullen, Shanmugan Uthamalingam, Gagandeep S Gurm, Shawn A. Gregory, Michael H. Picard

**Affiliations:** a Division of Cardiology, Massachusetts General Hospital, USA

**Keywords:** Transthoracic echocardiography, Wall motion, Ischemic heart disease

## Abstract

**Background:**

Our aim was to determine whether regional left ventricular (LV) function on a resting transthoracic echo (TTE) provides prognostic information in patients with varying degrees of ischemia on myocardial perfusion imaging.

**Methods:**

Between 2004 - 2009, we identified 503 patients (mean age 69 (SD 11); 79% male) with reversible ischemia on a myocardial SPECT scan who had a TTE within 30 days. We evaluated the rate of subsequent revascularization and death for all patients.

**Results:**

Following the SPECT scan and TTE, 246/503(49%) patients underwent revascularization, 64/503 (13%) patients died, 369 (73%) patients had a normal left ventricular ejection fraction (LVEF), 242 (48%) patients had a resting wall motion abnormality (WMA), 21/261 (8%) with no WMA died compared to 43/242 (18%) in patients with a WMA. In patients with a WMA (n = 242) there was no significant difference in mortality when comparing patients with small (< 6 segments) and large (> 6 segments) WMA (P = 0.44). In patients with moderate/severe ischemia, the presence of a resting WMA was associated with a higher mortality rate (18% v 7%; P = 0.005). In a multivariable model, LVEF (< 50%) was associated with a hazard ratio of 2.2 (P = 0.002, 95% CI 1.34 - 3.68) however, WMA and number of abnormal segments did not reach statistical significance.

**Conclusion:**

A resting wall motion abnormality in patients with moderate/severe ischemia is associated with a higher mortality compared to patients with mild ischemia on myocardial perfusion imaging. Regional left ventricular dysfunction unlike LVEF was not an independent predictor of mortality.

## Introduction

Global left ventricular systolic function as measured by left ventricular ejection fraction (LVEF) is a powerful predictor of outcome [1-5]. The prognostic value of induced wall motion abnormality in patients with coronary artery disease is well established [6-12]. Regional left ventricular systolic dysfunction as represented by the presence of a resting wall motion abnormality (WMA) is also associated with a higher cardiovascular event rate [11, 13-19]. However, it is unclear whether the prognostic value of the resting WMA relates to the severity of inducible myocardial ischemia or prior infarcts. Our aim was to determine whether regional left ventricular (LV) function on a resting transthoracic echo (TTE) could differentiate those with varying degrees of inducible ischemia and or provide prognostic information in these patients.

## Methods

Between 2004 - 2009, we identified 503 patients with reversible ischemia on a myocardial SPECT scan (Tc-99m sestamibi) who also had a TTE within 30 days of the scan as part of their routine care at the Massachusetts General Hospital. Using our institution’s computerized medical records we obtained baseline information regarding cardiac risk factors and medication. We also evaluated the rate of subsequent revascularization (percutaneous coronary intervention (PCI) or coronary artery bypass grafting (CABG) or death for all patients. The study was approved by the Institutional Review Board at Massachusetts General Hospital.

### Transthoracic echocardiography

Two dimensional transthoracic images were acquired in accordance with guidelines published by the American Society of Echocardiography. LVEF was determined using either a modification of the Quinones method [20] or the Biplane methods of discs [21]. The presence, severity and extent of WMA were interpreted by Cardiologists with level III training in echocardiography using a standard segmental model.

### Myocardial perfusion imaging

The SPECT- myocardial perfusion images (MPI) images using 99mTc-MIBI or thallium were acquired by a dual-head Siemens gamma camera (E-CAM or C-CAM; Siemens Medical Systems, Erlangen, Germany) in accordance with guidelines published by the American Society of Nuclear Cardiology [22]. Rest and stress myocardial perfusion images (MPI) were analyzed objectively based on the standard 17-segment model in a semi quantitative fashion using commercially available SPECT image analysis program (4DM, Ann Arbor, Michigan) by two physicians with level III training in nuclear cardiology. The parameters analyzed included summed rest score (SRS) reflecting the degree and severity of myocardial scar, summed stress score (SSS) reflecting both the degree and severity of myocardial ischemia and scar, summed difference score (SDS) (derived from SSS - SRS) reflecting the degree and extent of myocardial ischemia using a 5 point scoring system. Myocardial ischemia was categorized as mild when the SDS was 1 to 3, moderate when the SDS was 4 to 7, or severe when the SDS was > 7. LVEF was derived from gated stress SPECT-MPI, Analyses were performed independently blinded to the results of echocardiography and coronary angiography.

### Statistical analysis

Unless specified, data are presented as mean values ± SD. The chi-square test was used for categorical variables comparison. Univariate predictors of outcome were calculated with the Cox proportional hazards model. Multivariable analysis was performed using the Cox proportional hazards regression model to identify independent predictors of outcome. All analyses were performed using SPSS statistical software (SPSS Inc., Chicago, Illinois), and p values < 0.05 (two-tailed) were considered to be statistically significant. The primary end-point was all cause mortality.

## Results

There were 503 patients who met entry criteria (mean age 69 (SD 11); 79% male). The mean follow-up was 30 months. Baseline characteristics for all patients according to degree of ischemia are presented in Table 1, 369 (73%) patients had a normal LVEF, 242 (48%) patients had a WMA. Of the patients with a resting WMA, 126/240 (53%) had an EF ≤ 50%. In patients with no resting WMA 4/259, (2%) had an EF ≤ 50%.The extent of the resting WMA is summarized in Table 1. Following the SPECT scan and echo, 246/503 (49%) patients subsequently underwent PCI or CABG. A total of 64/503 (13%) patients died.

**Table 1:** Baseline Characteristics According to Degree of Ischemia (n = 503)

	Mild ischemia n = 230 (46%)	Mod /Sev ischemia n = 273 (54%)	P
Mean age (years) (SD)	69 (± 11)	68 (± 11)	0.18
Age > 65 years	148 (64%)	165 (60%)	0.41
Male gender	182 (79%)	215 (79%)	1.00
Hypertension	198 (86%)	225 (82%)	0.27
Hyperlipidemia	187 (81%)	212 (78%)	0.19
Diabetes	79 (34%)	85 (31%)	0.45
History of smoking	131 (57%)	168 (62%)	0.32
Previous MI	93 (40%)	113 (41%)	0.86
Previous CVA	31 (13%)	25 (9%)	0.15
Previous TIA	8 (3%)	8 (3%)	0.80
PVD	39 (17%)	56 (21%)	0.36
Chronic renal failure	19 (8%)	31 (11%)	0.30
Mean creatinine (SD)	1.3 mg/dL (± 0.8 mg/dL)	1.3 mg/dL (± 0.7 mg/dL)	0.87
Dialysis	5 (2%)	2 (1%)	0.26
Atrial fibrillation/flutter	47 (20%)	39 (14%)	0.07
Prior revascularization	109 (47%)	110 (40%)	0.13
Aspirin	175 (76%)	205 (75%)	0.75
Clopidogrel	49 (21%)	51 (19%)	0.50
Warfarin	33 (14%)	20 (7%)	0.01
Betablocker	157 (68%)	177 (65%)	0.39
ACE inh/ARB	125 (54%)	142 (52%)	0.59
Statin	160 (70%)	181 (66%)	0.44
Diuretic (loop or thiazide)	70 (30%)	86 (32%)	0.85
Aldosterone antagonist	6 (3%)	6 (2%)	0.78
Calcium channel blocker	44 (19%)	61 (22%)	0.44
Digoxin	16 (7%)	10 (4%)	0.11
Echo			
EF (mean, SD)	58% (14)	58% (13)	0.78
EF > 50%	168 (73%)	201 (74%)	0.68
WMA	115 (50%)	127 (47%)	0.47
≤ 3 segments	25 (11%)	21 (9%)	0.33
≤ 6 segments	37 (16%)	46 (17%)	0.59
> 6 segments	36 (16%)	42 (15%)	0.78
Diffusely hypokinetic	17 (7%)	18 (7%)	1.00
Nuclear			
Summed Rest Score	16 (± 9)	12 (± 7)	< 0.001
Summed Stress Score	19 (± 9)	18 (± 7)	0.53
Summed Difference Score	3 (± 3)	7 (± 4)	< 0.001
Revascularization	79 (34%)	167 (61%)	< 0.001
Death	31 (13%)	33 (12%)	0.69

### Degree of ischemia

A total of 230 (46%) patients had mild ischemia and 273 (54%) had moderate/severe ischemia on a SPECT scan. The summed rest score (SRS) for patients with mild ischemia was 16.2 (± 8.6) compared to 12.0 (± 7.2) for patients with moderate/severe ischemia (P < 0.001). The summed stress score (SSS) for patients with mild ischemia was 18.9 (± 9.0) compared to 18.4 (± 7.1) for patients with moderate/severe ischemia (P = NS). The summed difference score (SDS) for patients with mild ischemia was 2.8 (± 2.9) compared to 6.6 (± 3.9) for patients with moderate/severe ischemia (P < 0.001). There was no significant difference in LVEF or WMA between the 2 groups of ischemia. A total of 219/503 (44%) patients had a history of previous revascularization (47% in mild ischemia group V 40% in the moderate/severe ischemia group). The rate of subsequent revascularization was significantly higher in the moderate/severe ischemia group (61% v 34%; P < 0.001). No significant difference in mortality was observed between the 2 groups of ischemia.

### Degree of ischemia and resting WMA

Groups were then analyzed according to degree of ischemia and the presence or absence of a resting WMA. Table 2 summarizes the rates of revascularization and death in these 4 groups. As a substantial number of patients had a history of prior revascularization, Table 3 summarizes rates of subsequent revascularization and death in a subset of patients with no prior history of revascularization.

**Table 2:** Summary of Revascularization and Outcomes According to Degree of Ischemia and WMAs (n = 503)

	Mild ischemia and no WMA (n = 115)	Mild ischemia and WMA (n = 115)	P value
Prior revascularization	50 (43%)	59 (51%)	0.29
Revascularization	47 (41%)	32 (28%)	0.05
Death	11 (10%)	20 (17%)	0.12
			
	Moderate/severe ischemia and no WMA (n = 146)	Moderate/severe ischemia and WMA (n = 127)	P value
Prior revascularization	45 (31%)	65 (51%)	0.001
Revascularization	98 (68%)	69 (54%)	0.03
Death	10 (7%)	23 (18%)	0.005

Eighteen patients who were revascularized subsequently died.

**Table 3:** Summary of Revascularization and Outcomes According to Degree of Ischemia and WMAs in a Subset of Patients With no History of Prior Revascularization (n = 284)

	Mild ischemia and no WMA on Echo (n = 65)	Mild ischemia and WMA on Echo (n = 56)	P value
Revascularization	30 (46%)	20 (36%)	0.27
Death	6 (9%)	10 (18%)	0.19
			
	Moderate/severe ischemia and no WMA on Echo (n = 100)	Moderate/severe ischemia and WMA on Echo (n = 63)	P value
Revascularization	76 (76%)	41 (65%)	0.22
Death	6 (6%)	12 (19%)	0.01

Ten patients were revascularized and subsequently died.

Patients with no resting WMA had a better prognosis compared with patients with a resting WMA, 21/261 (8%) with no resting WMA died compared to 43/242 (18%) in patients with a resting WMA. In patients with moderate/severe ischemia, the presence of a resting WMA was associated with a higher mortality rate (18% v 7%; P = 0.005). There was no significant difference in mortality in the group with mild ischemia regardless of the presence or absence of a resting WMA. In patients with a resting WMA (n = 242) there was no significant difference in mortality when comparing patients with less or more than 6 segments involved (P = NS). Table 4 shows the extent of wall motion abnormality on echo represented by the number of abnormal segments and all cause mortality.

**Table 4:** A. Extent of WMA on Echo According to Segmental Abnormalities and All Cause Mortality (n = 503) P = 0.01 (ANOVA); B. Extent of WMA on Echo According to Segmental Abnormalities and All Cause Mortality in Patients With Mild Ischemia (n = 230) P = 0.39 (ANOVA); C. Extent of WMA on Echo According to Segmental Abnormalities and All Cause Mortality in Patients With Moderate/Severe Ischemia (n = 273) P = 0.005 (ANOVA)

A	0 (n = 261)	≤ 3 (n = 46)	≤ 6 (n = 83)	> 6 (n = 78)	Diffuse (n = 35)
Dead	21 (8%)	6 (13%)	13 (16%)	16 (21%)	8 (23%)
Alive	240 (92%)	40 (87%)	70 (84%)	62 (79%)	27 (77%)
					
B	0 (n = 115)	≤ 3 (n = 25)	≤ 6 (n = 37)	> 6 (n = 36)	Diffuse (n = 17)
Dead	11 (10%)	5 (20%)	6 (16%)	5 (14%)	4 (24%)
Alive	104 (90%)	20 (80%)	31 (84%)	31 (86%)	13 (76%)
					
C	0 (n = 146)	≤ 3 (n = 21)	≤ 6 (n = 46)	> 6 (n = 42)	Diffuse (n = 18)
Dead	10 (7%)	1 (5%)	7 (15%)	11 (26%)	4 (22%)
Alive	136 (93%)	20 (95%)	39 (85%)	31 (74%)	14 (78%)

In the group with mild ischemia, the LVEF was similar regardless of the presence of a resting WMA. Furthermore, there was no significant difference in mortality between the two groups. Only 2% of patients with mild ischemia and no resting WMA had an LVEF < 50%. In the group with moderate/severe ischemia, the LVEF was lower in patients with resting WMA as was the mortality rate. Only 1% of patients with moderate/severe ischemia and no resting WMA had LVEF < 50%.

### Univariate analysis

Table 5 summarizes the predictive value of variables for overall mortality in a univariate analysis. Age, history of hypercholesterolemia, cerebrovascular accident (CVA), peripheral vascular disease (PVD), creatinine, LVEF, presence of resting WMA and number of abnormal segments were all predictors of mortality. Sex, history of hypertension, diabetes, smoking, degree of ischemia, SDS, and prior revascularization were not predictors of mortality in this study group.

**Table 5:** Univariable Analysis

	HR	P value	95% CI
Age	1.053	< 0.001	1.027 - 1.081
Sex	1.242	0.113	0.950 - 1.625
Hypertension	0.919	0.639	0.646 - 1.308
Hypercholesterolemia	1.362	0.021	1.049 - 1.768
Diabetes	0.847	0.191	0.660 - 1.087
CVA	0.641	0.003	0.480 - 0.856
PVD	0.649	0.001	0.503 - 0.838
History of smoking	0.868	0.584	0.523 - 1.440
Prior revascularization	0.896	0.660	0.548 - 1.463
Creatinine	1.293	0.003	1.093 - 1.529
Echo EF >/< 50%	0.339	< 0.001	0.208 - 0.554
Resting WMA	0.459	0.003	0.272 - 0.774
Number of abn segments	1.905	0.013	1.148 - 3.162
Degree of ischemia	1.102	0.437	0.862 - 1.409
SDS	1.003	0.915	0.943 - 1.067

### Multivariable analysis

Independent predictors of mortality included age (HR 1.0, 95% CI 1.02 - 1.08; P = 0.001), creatinine (HR 1.3, 95% CI 1.08 - 1.67; P = 0.008), hypercholesterolemia (HR 1.7, 95% CI 1.03 - 2.98., P = 0.037), CVA (HR 2.1, 95% CI 1.19 - 3.81, P = 0.011) and PVD (HR 1.7, 95% CI 1.0 - 2.83, P = 0.05). LVEF < 50% was associated with a HR 2.2 (95% CI 1.34 - 3.68; P = 0.002), however, the presence of a resting WMA, extent of WMA categorized by number of segments (>/< 6) and summed stress score were not independent predictors of mortality (Table 6).

**Table 6:** Multivariable Analysis (Forward Conditional Model)

	HR	P value	95% CI
Age	1.0	0.001	1.02 - 1.08
Creatinine	1.3	0.008	1.08 - 1.67
Ejection Fraction < 50%	2.2	0.002	1.34 - 3.68
Hypercholesterolemia	1.7	0.037	1.03 - 2.98
CVA	2.1	0.011	1.19 - 3.81
PVD	1.7	0.05	1.00 - 2.83

## Discussion

Our aim was to determine whether regional left ventricular function, on a resting echo, provided incremental prognostic information in patients with varying degrees of myocardial ischemia. In this study of 503 subjects, there was no significant difference in LVEF and the presence of a resting WMA in patients with mild and moderate/severe ischemia, 64 (13%) of patients died after a mean follow-up of 30 months. The presence of a resting WMA in patients with moderate/severe ischemia was associated with a higher mortality. For those with mild ischemia the presence of a WMA was not associated with differences in outcome.

It is well established that LVEF is an excellent prognostic marker in patients post myocardial infarction [1-5]. Regional left ventricular function provides additional prognostic information in patients post myocardial infarction [17, 23], and in patients with heart failure [18]. Although the presence of a resting WMA generally represents prior infarction, stunned or hibernating myocardium, it may also be found in individuals without coronary artery disease [13]. In a study of 2864 subjects without clinically evident cardiovascular disease, 140 pts (5%) had a resting wall motion abnormality. The cumulative incidences of combined fatal and nonfatal cardiovascular mortality were 2.5 to 3 fold higher in those with segmental wall motion abnormalities compared to those with no resting WMA [13]. In populations without clinical evidence of cardiovascular disease resting WMA can predict abnormal perfusion and ischemia [24]. In patients without known coronary artery disease referred for evaluation of ischemia, 21% had WMA detected on baseline TTE. The prevalence of myocardial perfusion abnormalities was higher in patients with resting WMA (75% v 25%). The presence of a resting WMA was an independent predictor of ischemic response [24].

Some studies have shown that in certain subgroups of patients, resting WMA is a more powerful predictor of cardiovascular events than LVEF in patients after myocardial infarction [17, 19]. In a study of 233 patients with diabetes, mean LVEF 52% and negative dobutamine stress echo, resting WMA, unlike LVEF was an independent predictor of future events including death, non-fatal ST-elevation MI, non-ST elevation MI (NSTEMI) and late (> 6 months) revascularization [16]. In a study of patients with end stage renal failure the presence of both a resting WMA and inducible ischemia on dobutamine stress echo were associated with a higher rate of adverse events. The presence of a fixed WMA alone was not, however, a predictor of major adverse cardiac events (MACE) in this cohort of subjects [25].

Left ventricular global and regional function is closely related and most patients with significant WMAs have a reduced LVEF [26]. LVEF however, reflects global function and hypercontractile segments may offset hypokinetic segments thereby preserving overall LV systolic function. Some studies have reported that resting WMA is a more powerful predictor of outcome [17] whereas others report incremental prognostic information using both resting WMA and LVEF [18]. In our study, 53% with a resting WMA had an EF ≤ 50%, whereas 2% of patients with no resting WMA had an EF ≤ 50%. In all patients, LVEF, the presence of a resting WMA and the number of abnormal segments were all univariate predictors of death during a mean follow-up period of 30 months.

From our retrospective study, it is not possible to determine why patients with moderate/severe inducible ischemia and a WMA on resting echo have a worse prognosis than those without a resting WMA. One could speculate several possible explanations. It is possible that those with a resting WMA not only have had prior infarction but also more severe coronary artery disease. One would expect the resting WMA to impact LVEF which is a strong marker of outcome as seen in our multivariate analysis and prior studies [26].

### Limitations of this study

This is a retrospective study with its inherent limitations. The study group represents a select cohort of moderate to high risk patients with documented ischemia on SPECT. Consequently, the results may not be generalizable to other populations.

### Summary

The presence of a resting WMA on TTE in patients with moderate and severe ischemia by myocardial perfusion imaging is associated with a higher mortality compared to patients with no resting WMA and moderate/severe ischemia. Regional left ventricular dysfunction, however, unlike LVEF was not an independent predictor of mortality. LV dysfunction whether global or regional is a powerful prognostic marker. Early identification of these high risk patients allows optimization of medical therapy +/- referral for revascularization.
